# Research progress of exosomes in the angiogenesis of digestive system tumour

**DOI:** 10.1007/s12672-024-00879-4

**Published:** 2024-02-11

**Authors:** Yuan Liu, Hao Wu, Yaodong Sang, Wei Chong, Liang Shang, Leping Li

**Affiliations:** 1 Department of Gastroenterological Surgery, Shandong Provincial Hospital, Shandong University, Jinan, 250021 Shandong China; 2 Department of General Surgery, Peking Union Medical College, Peking Union Medical College Hospital, Chinese Academy of Medical Sciences, Beijing, 100730 China; 3 Department of Gastrointestinal Surgery, Key Laboratory of Engineering of Shandong Province, Shandong Provincial Hospital Affiliated to Shandong First Medical University, Shandong Provincial Hospital, Jinan, 250021 China; 4 Department of Gastrointestinal Surgery, Medical Science and Technology Innovation Center, Shandong First Medical University & Shandong Academy of Medical Sciences, Shandong Provincial Hospital, Cheeloo College of Medicine, Shandong University, Jinan, 250021 China

**Keywords:** Exosome, Angiogenesis, Antiangiogenic therapy, Digestive system tumour

## Abstract

Malignant tumours of the digestive system cover a wide range of diseases that affect the health of people to a large extent. Angiogenesis is indispensable in the development, and metastasis of tumours, mainly in two ways: occupation or formation. Vessels can provide nutrients, oxygen, and growth factors for tumours to encourage growth and metastasis, so cancer progression depends on simultaneous angiogenesis. Recently, exosomes have been proven to participate in the angiogenesis of tumours. They influence angiogenesis by binding to tyrosine kinase receptors (VEGFR)-1, VEGFR-2, and VEGFR-3 with different affinities, regulating Yap-VEGF pathway, Akt pathway or other signaling pathway. Additionally, exosomes are potential therapeutic vectors that can deliver many types of cargoes to different cells. In this review, we summarize the roles of exosomes in the angiogenesis of digestive system tumours and highlight the clinical application prospects, directly used as targers or delivery vehicles, in antiangiogenic therapy.

## Background

The digestive system is one of the most important systems in our body, and related tumours frequently occur [1]. Digestive system malignant tumours can be classified into oesophageal, gastric, small intestinal, colon, rectal, appendiceal, anal, hepatic and pancreatic cancer based on the organ from which the abnormal cell proliferation is derived [2]. These tumours cause adverse effects on the physical and mental health of high-risk individuals [3, 4]. According to the report released by the International Agency for Research on Cancer, for both sexes combined, four digestive system cancer types are in the top 10 cancer types for worldwide incidence (23.4% of total cases) and five are in the top 10 for worldwide mortality (35.6% of total cases) [5]. Due to the lack of typical early symptoms, early detection is often difficult and patients often miss the opportunity for optimal treatment [6, 7]. Although endoscopy, cholangiopancreatography and needle aspiration techniques have improved, the invasiveness, uncertainty of operation and high cost restrict large-scale screening [8, 9]. At present, serum carcinoembryonic antigen (CEA), Carbohydrate antigen 19–9 (CA19-9), and Carbohydrate antigen 125 (CA125) are normally used in clinical diagnosis, but both their sensitivity and specificity are low [10–12]. Therefore, noninvasive and highly accurate tumour biomarkers are urgently needed to strongly support the screening of digestive system tumours, even in the early stages.

Exosomes refer to a specific subtype of secreted derived vesicles with a lipid bilayer membrane structure, a size of approximately 30–150 nm in diameter [13]. Exosomes are secreted by almost all human cells and have been found in numerous biological fluids, such as sperm, blood (serum and/or plasma), breast milk, urine, amniotic fluid, saliva, cerebrospinal fluid, nasal secretions, bronchoalveolar lavage, bile, synovial fluid, malignant effusions, pleural effusions and ascites [14]. In both physiological and pathological processes, exosomes play a crucial role in cell–cell communication [15]. Due to their special structural characteristics and capacity to carry many cargos, [16] exosomes have become hot research topics in recent years.

**Fig. 1:**
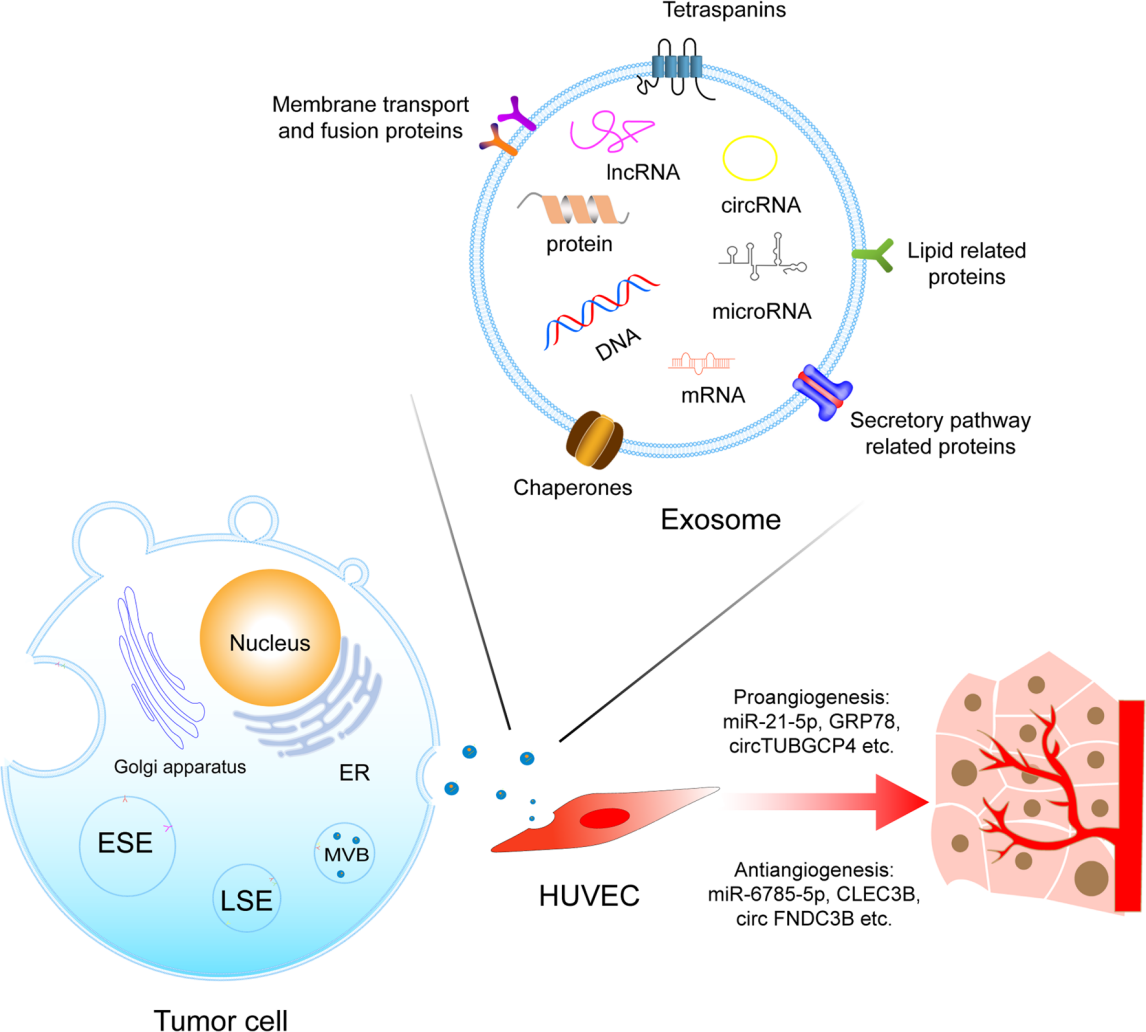
Tumour-derived exosomes are generated through ESE, LSE, and ILVs in MVBs. After being secreted, exosomes can participate in the pro- and anti-angiogenesis of cancer by the cargos. *ESE* early-sorting endosome, *LSE* late-sorting endosome, *ILVs* intraluminal vesicles, *MVBs* multivesicular bodies, *HUVEC* human umbilical vein endothelial cell

Vessels can provide nutrients, oxygen, and growth factors for tumours to encourage growth and metastasis, so cancer progression depends on simultaneous angiogenesis, which means tumours and angiogenesis are supplementary to each other [17]. The process by which new capillaries arise from preexisting blood vessels is named angiogenesis [18]. The balance between pro- and antiangiogenic factors controls the process of angiogenesis [19]. Tumour angiogenesis and pathological activation of the endothelium, tumour vessel leakiness and hypoxia-induced apoptosis/necrosis in the tumour core have a vital role in recruiting and activating many more inflammatory cells, such as lymphocytes, neutrophils, macrophages and mast cells [20]. These infiltrating immune cells secrete growth and angiogenic factors into the microenvironment to enhance cancer growth and subsequent resistance to therapy [20]. The tumour microenvironment (TME) is a dynamic environment in which multiple components interact and influence each other to regulate the process of the disease [21]. Exosomes participate in the angiogenesis of cancer by influencing endothelial cells through growth factors or direct interactions [22] (Fig. 1). This review focuses on exosomes in the context of tumour angiogenesis in digestive system tumours and describes their therapeutic properties as targets for future antiangiogenic drugs.

### Exosome biogenesis, composition and regulation

Exosomes are a subtype of small extracellular vesicles (sEVs) [23]. Exosomes are generated through the early-sorting endosome (ESE), late-sorting endosome (LSE), and intraluminal vesicle (ILVs) phases in multivesicular bodies (MVBs) before they exit the cell [24]. ESEs arise from the inward budding of the cell’s plasma membrane with the help of the trans-Golgi network and endoplasmic reticulum [25]. Then, they mature into LSEs and eventually invaginate from the endosomal limiting membrane to form MVBs, which contain several ILVs (future exosomes) [26]. MVBs are finally either sent to lysosomes to be degraded or fused with the cell plasma membrane to release the contained ILVs as exosomes (Fig. 1) [25]. The mechanisms of exosome biogenesis and formation and vesicle scission are diverse. The endosomal sorting complex needed for transport (ESCRT)-dependent and ESCRT-independent machinery are widely studied [27, 28]. Other factors, such as Vps60 [29], IST1 [30], and synaptotagmin 7 (SYT7) [31], have also been reported.

The contents of exosomes are complex and include microRNAs, circular RNAs, long noncoding RNAs (lncRNAs), DNA fragments, proteins, lipids and metabolites (Fig. 1) [32, 33]. These cargoes determine the function of exosomes to a great degree [34, 35]. Exosomes modulate the tissue microenvironment by mediating autocrine, juxtacrine and paracrine interactions, and they also participate in the suppression of antitumour immune responses, cancer angiogenesis, and tumour progression [36]. Exosome uptake by target cells is processed by lipid raft-mediated endocytosis, receptor-mediated endocytosis, phagocytosis, micropinocytosis and membrane fusion [37]. However, the detailed mechanisms remain unclear [32].

Exosomes need to be isolated from biological fluids for various studies [38–40]. Until now, differential ultracentrifugation has been the most commonly used method for exosome separation [41–43]. Density gradient centrifugation, filtration, size-exclusion chromatography, and liquid chromatography techniques have also been applied [44]. The identification of exosomes is similarly important. Since exosomes are a type of sEV, they are suitable for routine detection and analysis [45]. Specifically, they are positive for at least one transmembrane/lipid-bound protein (usually CD9, CD63, CD83 and integrin), and at least one cytosolic protein can be recovered from EVs (usually ALIX, TSG101, syntenin and HSP70) [46, 47]. At the same time, the levels of at least one negative protein, such as albumin, lipoproteins, and ribosomal proteins, should also be determined [46, 47]. In addition, analysis of functional proteins, such as histones, cytochrome C, calnexin or Grp94, is needed for specific sEVs [46, 47]. Therefore, western blotting, fluorescence microscopy, flow cytometry and mass spectrometry are helpful to identify these proteins [48]. Every technique has both advantages and disadvantages with regard to sensitivity, specificity, time and cost [49]. Combinations of methodologies may be increasingly accepted for the absolute isolation of exosomes.

### Mechanism of angiogenesis

The formation of cancer blood vessels involves two aspects. One is the incorporation of host blood vessels into tumours, and the other is the ability of cancer cells to express the endothelial cell (EC) phenotype and form similar vessels [50]. At the initial stage of tumour progression, vessels may not be essential, and tumour cells acquire oxygen, nutrients, and growth factors from nearby vascular tubes through diffusion [51–53]. Alter the phrase. Although it is generally correlated with increased necrosis, the requirement for angiogenesis is more correlated with the oxygen gradient within the tumor, with tumor inner cells exposed to lower levels of oxygen resulting in necrosis [54]. However, newly generated vasculature in tumours is variable in size, shape, architecture, and arrangement compared to normal vessels [55]. These irregular, immature, tortuous, and distended vessels are produced because extracellular signals, such as hypoxia, low pH, a deregulated and disorganized extracellular matrix (ECM), mechanical stresses, and soluble mediators released by surrounding tumour and stromal cells, destroy the balance of pro- and antiangiogenic factors [56]. At least four mechanisms underlie angiogenesis, namely, sprouting angiogenesis (SA), splitting angiogenesis, also known as intussusceptive angiogenesis (IA), vessel co-option (VC) and vascular mimicry (VM) [57]. SA depends on angiogenic factors, such as growth factors, chemokines, angiopoietins, endostatin, interferons, and NO [58]. Angiogenic factors and the absence of blood flow mediate blood vessel destabilization to create conditions for a new sprout. Then, tip and stalk cells are differentiated and combine to sprout. Next, the apical membrane of stalk cells forms and extends new lumenized vascular tubes through tight and adhering connections with neighbouring sprouts or blood vessels [59]. SA completes when blood vessels stabilize, mature and prune [60]. During IA, intussusceptive pillars are generated from contacted endothelial cells that extend processes into the vascular lumen and fuse with the assistance of pericytes, fibroblasts, and other supporting cells to split vessels [61]. For VC, tumours hijack preexisting blood vessels of the nonmalignant tissue that they colonize by migrating along the abluminal surface of preexisting vessels and/or infiltrating the tissue space between pre-existing vessels [62]. It is described that cancer cells can utilize their capacity for epithelial-to-mesenchymal transition and acquire cancer stem cell-like behaviour to assume an endothelial-like phenotype, ultimately forming matrix-embedded vascular structures, including plasma and blood cells. This process is called VM [63, 64].

Although these four mechanisms of angiogenesis are known, various regulators and signalling pathways involved in the mechanisms are still being researched [65–67]. The classical factor is vascular endothelial growth factor A (VEGF-A). It is in a family that includes VEGF-A, VEGF-B, VEGF-C, VEGF-D, virally encoded VEGF-E, and placental growth factor (PLGF). They influence angiogenesis by binding to tyrosine kinase receptors (VEGFR)-1, VEGFR-2, and VEGFR-3 with different affinities [68]. The matrix metalloproteinase (MMP) family can modulate the dynamic remodelling of the ECM, the VEGF pathway and vascular permeability through different signalling axes [69–71]. For example, the MMP-1/protease-activated receptor-1 (PAR1) signalling axis induces vascular permeability to participate in tumour angiogenesis [72]. Under similar conditions, Forkhead Box M1 D (FOXM1D) upregulated VEGFA expression by binding to pyruvate kinase M2 (PKM2) and the NF-κB subunits p65 and p50 to promote their nuclear translocation [73]. Furthermore, FOXM1D increased the release of VEGFA and exosomes by interacting with VPS11 [73]. The platelet-derived growth factor (PDGF) family consists of four heparin-binding polypeptide growth factors (A, B, C, and D). It promotes vessel maturation, the recruitment of pericytes and VEGF upregulation [74]. The PDGF-B/PDGFRβ axis has been extensively explored [75]. The fibroblast growth factor (FGF) family has garnered interest. It consists of 22 members, most of which interact with the tyrosine kinase receptors FGFR-1, FGFR-2, FGFR-3, FGFR-4. The FGF-dependent control of c-MYC expression regulates levels of the glycolytic enzyme hexokinase 2 (HK2) to participate in vascular growth and development [76]. Angiopoietin is another noteworthy angiogenic factor. Angiopoietins 1–4 act through the tyrosine kinase receptors Tie-1 and Tie-2. Ang1 inhibits Forkhead Box O (Foxo), a negative regulator of angiogenesis, through Akt activation to produce vessels [77]. Ang2 can induce endothelial cell migration and sprout formation by binding to integrins in Tie2-dependent or Tie2-independent signalling [78]. In addition, many antiangiogenetic factors, such as thrombospondin-1 (TSP-1), vasostatin, and interferon, are being studied. The imbalance of pro- and antiangiogenic factors is also influenced by environmental elements, such as hypoxia, cellular nutrient deficiency, hypoglycaemia, and metabolic acidosis, which often act at the gene level [53].

Recently, tumour-derived exosomes (TEX) have been shown to markedly affect angiogenesis [79–81]. Data show that miR-23a from hypoxic tumour cell colonies increased the expression levels of angiogenic marker genes, such as VEGF, VEGFR2, and MMP9, by modulating the levels of SIRT1 [82]. Both in vitro and in vivo studies revealed that TEX carrying enzymatically active CD39/CD73 and adenosine (ADO) enhanced the secretion of angiogenic factors by directly interacting with ADO receptors or promoting A2BR-mediated polarization of macrophages towards an M2-like phenotype [83]. In addition, experiments using hypoxic papillary thyroid cancer cells showed that exosomal miR-181a promoted angiogenesis by downregulating histone-lysine N-methyltransferase-3 (MLL3) and disheveled binding antagonist of beta-catenin 2 (DACT2), as well as activating the YAP-VEGF pathway [84].

### Exosomes in angiogenesis of digestive system tumours

Numerous studies have convincingly proven that TEX induces molecular and genetic programs in EC changes and thus promotes the process of angiogenesis [85]. Most studies verified the relationship between exosomes and angiogenesis by coculturing exosomes with human umbilical vein endothelial cells (HUVESs) in vitro and creating an in vivo nude mouse xenograft model [86]. A list of the tumour-derived exosome cargoes involved in the stimulation of angiogenesis according to the several mentioned studies is shown in Table 1.

**Table 1:** Exosomes in angiogenesis of digestive system tumours

Cancer	Function	Type	Contents	Reference
Oesophageal cancer	Proangiogenesis	lncRNA	FAM225A	[88]
		microRNA	miR-21-5p	[90]
			miR-301a-3p	[91]
	Antiangiogenesis	microRNA	miR-154-5p	[93]
Gastric cancer	Proangiogenesis	protein	YB-1	[96]
		protein	GRP78	[101]
		circular RNA	circSHKBP1	[95]
		circular RNA	circ29	[98]
		microRNA	miR-23a	[94]
		microRNA	miR-10a-5p	[99]
		microRNA	miR-519a-3p	[187]
		ncRNA	X26nt	[97]
	Antiangiogenesis	microRNA	miR-6785-5p	[188]
Colorectal cancer	Proangiogenesis	protein	Angiopoietin-like 7(ANGPTL7)	[104]
		protein	Wnt4	[106]
		protein	Glucose-regulated protein 78 (GRP78)	[107]
		protein	dipeptidyl peptidase IV (DPP4)	[115]
		protein	growth/differentiation factor 15 (GDF15)	[116]
		circular RNA	circTUBGCP4	[117]
		circular RNA	circ_0007334	[114]
		microRNA	miR‑1246	[105]
		microRNA	miR-25-3p	[108]
		microRNA	miR-1229	[109]
		microRNA	miR-92a-3p	[110, 111]
		microRNA	miR-183-5p	[112]
		microRNA	miR-21-5p	[113]
		microRNA	miR-135b-5p	[118]
	Antiangiogenesis	circular RNA	circ FNDC3B	[120]
		microRNA	miR-126	[121]
		microRNA	miR-125a-3p	[121]
		microRNA	miR-125a-5p	[121]
		microRNA	miR-34a	[122]
Hepatic cancer	Proangiogenesi	protein	lysyl oxidase-like 1–4 (LOXL1–4)	[130]
		protein	Angiopoietin-2 (ANGPT2)	[132]
		protein	RAB13	[140]
		lncRNA	lncRNA H19	[123]
		lncRNA	LINC00161	[133]
		lncRNA	small nucleolar RNA host gene 16 (SNHG16)	[139]
		circular RNA	circRNA-100,338	[131]
		circular RNA	circCMTM3	[138]
		microRNA	miR-32-5p	[124]
		microRNA	miR-210	[125]
		microRNA	miR-155	[126]
		microRNA	miR-21	[127]
		microRNA	miR-378b	[136]
		microRNA	miR-1290	[137]
	Antiangiogenesis	protein	C-Type Lectin Domain Family 3 Member B (CLEC3B)	[141]
		microRNA	miR-200b-3p	[142]
		microRNA	miR-3682-3p	[143]
Pancreatic cance	Proangiogenesis	protein	annexin A1 (ANXA1)	[144]
		lncRNA	lncRNA SNHG11	[146]
		lncRNA	lncRNA UCA1	[148]
		microRNA	miR-27a	[145]
		microRNA	miR-30b-5p	[149]
		microRNA	miR-210	[150]
		microRNA	miR-501-3p	[152]
		microRNA	miRNAs 155-5p and 221-5p	[153]
	Antiangiogenesis	microRNA	miR-29b	[151]

### Oesophageal cancer

Several studies have described the role of exosomes in the angiogenesis of oesophageal cancer. Yu Mao et al. performed a Matrigel tube formation assay, examined the recruitment of vasculature into subcutaneously implanted Matrigel plugs containing exosomes and concluded that exosomes promote endothelial cell recruitment and vascular organization both in vitro and in vivo, especially hypoxic exosomes [87]. Recently, the exosome-mediated transfer of lncRNA FAM225A was revealed to upregulate NETO2 and FOXP1 expression by sponging miR-206 and accelerating oesophageal cancer progression and angiogenesis [88]. Furthermore, poly (A) binding protein cytoplasmic 1 (PABPC1) activates the IFN/IFI27 signalling pathway to enhance oesophageal squamous cell carcinoma (ESCC) proliferation; at the same time, PABPC1/IFI27 can increase exosomal miRNA-21-5p to promote angiogenesis by inhibiting CXCL10 [89]. Moreover, a review has shown that miRNA-21-5p accelerates angiogenesis by activating programmed cell death 4 and downregulating the signalling pathway or the PTEN (phosphatase and tensin homologue)/Akt signalling pathway [90]. ESCC-derived exosomal miR-301a-3p induced macrophage polarization into the M2 type via the inhibition of PTEN and activation of the PI3K/AKT signalling pathway, subsequently promoting angiogenesis via the secretion of VEGFA and MMP9 [91]. In addition to proangiogenesis, the antiangiogenesis of exosomes in ESCC cells has also been described [92]. Specifically, miR-154-5p overexpression in exosomes inhibited angiogenesis in vitro, as indicated by the inhibition of tube formation in HUVECs. This inhibition may have been mediated by a reduction in kinesin family member 14 (KIF14) expression in ESCC cells via the direct targeting of the KIF14 3ʹUTR. [93] Additionally, many exosomes are secreted by oesophageal cancer cells, but whether they play key roles in the formation of vessels still needs rigorous experiments.

### Gastric cancer

Angiogenesis has been researched in gastric cancer (GC). Evidence has indicated that exosomal miR-23a is a proangiogenic factor because it negatively regulates PTEN, which is a tumour angiogenesis suppressor [94]. A series of analysis assays showed that exosomal circSHKBP1 enhanced VEGF mRNA stability and induced VEGF translation by decreasing miR-582-3p to increase HUR expression [95]. YB-1 is considered to be conducive to the neovasculature of GC via exosomes, which may directly or indirectly influence proangiogenic factors [96]. Xiaocui Chen et al. revealed that exosomal X26nt, which is a 26-nt-long ncRNA spliced from inositol- requiring enzyme 1 alpha (IRE1α)- induced unspliced XBP1, directly combined with the 3′UTR of VE- cadherin mRNA in HUVECs to enhance vascular permeability and accelerate angiogenesis [97]. A recently published study showed that exosomal circ29 in GC regulated angiogenesis via the VEGF pathway as a sponge of miR-29a [98]. Subsequently, miR-10a-5p downregulated zinc finger MYND-type containing 11 (ZMYND11) to enhance the viability and migration of HUVECs, which are packaged into GC cell-derived exosomes [99]. A novel study revealed that circFCHO2 was overexpressed in the serum exosomes of GC patients, which might enhance the progression of GC by activating the JAK1/STAT3 signalling pathway by sponging miR-194-5p [100]. In another study, exosomal glucose-regulated protein 78 (GRP78) was found to promote the TME and induce angiogenesis. [101] GC-derived exo-miR-519a-3p are mainly accumulates in the liver and is internalized by intrahepatic macrophages, activating the MAPK/ERK pathway by targeting DUSP2, thereby causing M2-like polarization of macrophages [102]. M2-like polarized macrophages induce angiogenesis and promote intrahepatic premetastatic niche formation to accelerate liver metastasis in gastric cancer patients [103]. These exosomal cargoes, which promote or suppress GC angiogenesis, provide new treatment options, but their mechanism should be further investigated.

### Colorectal cancer

Cancer of the colon, retum and anus are classified as colorectal cancer. Experimental evidence supports that angiopoietin-like 7 (ANGPTL7) plays a role in proangiogenesis and vascularization in colorectal cancer, and this process is partially associated with exosomes [104]. Colorectal cancer (CRC) cells can transfer angiogenesis‑promoting miR‑1246 to endothelial cells via exosome transfer [105]. By targeting promyelocytic leukaemia (PML) protein, Smad 1/5/8 signalling is activated, enhancing angiogenesis [105]. Wnt4 loaded in cancer-derived exosomes is conducive to the angiogenesis of cancer via the β-catenin signalling pathway [106]. GRP78 is a known proangiogenic factor in CRC that can be secreted into the microenvironment by tumour cells through the exosome pathway [107]. Moreover, miR-25-3p, which is carried by exosomes derived from CRC, reportedly increases the expression of VEGFR2 and decreases the levels of ZO-1, occludin and claudin-5 in endothelial cells by targeting Krüppel-like factor 2 (KLF2) and Krüppel-like factor 4 (KLF4), consequently promoting vascular permeability and angiogenesis [108]. Exosomal miR-1229 could facilitate angiogenesis by repressing the protein expression of HIPK2 to activate the VEGF pathway [109]. The pro-angiogenic function of miR-92a-3p is attributed to the decreasing expression of at least two target genes, Dickkopf-3 (Dkk-3) [110] and claudin-11(CLDN11), which may induce partial endothelial-to mesenchymal transition [111]. A cell coculture model was used to show that exosomes loaded with miR-183-5p promoted the tube formation abilities of CRC by inhibiting FOXO1 [112]. Compelling evidence has demonstrated that exosomal miR-21-5p suppressed Krev interaction trapped protein 1 (KRIT1) in recipient HUVECs and subsequently activated the β-catenin signalling pathway and increased their downstream targets VEGFa and Ccnd1, enhancing angiogenesis and vascular permeability in CRC [113]. Exosomal circ_0007334 in CRC cells was found to directly bind to miR-577 and target Krüppel-like Factor 12 (KLF12) to promote angiogenesis and tumour growth [114]. Exosomes also paly roles in drug-resistant colon cancer cells. Research has shown that dipeptidyl peptidase IV (DPP4)-enriched exosomes, which are secreted by 5-fluorouracil-resistant colon cancer cells, mediate angiogenesis by increasing the expression and secretion of periostin (POSTN) (a proangiogenic extracellular matrix protein) via Twist1 nuclear translocation or activating the Smad signalling pathway [115]. However, exosomal growth/differentiation factor 15 (GDF15) plays an essential role in the effects of angiogenesis, which increases POSTN levels by inhibiting the Smad signalling pathway [116]. Exosomal circTUBGCP4 upregulated PDK2 to activate the Akt signalling pathway by sponging miR-146b-3p, which causes vascular endothelial cell tipping to promote angiogenesis and tumour metastasis [117]. In addition to TEX, cancer-associated fibroblast (CAF)-derived exosomes (CAF-exos) can also transmit microRNAs (miRNAs) to CRC. Recently, miR-135b-5p, which is transported by CAF-exos, was reported to downregulate thioredoxin-interacting protein (TXNIP) to promote angiogenesis [118]. Experiments have also confirmed that CAF-Exos deliver VEGFA to promote the viability, apoptosis, DDP resistance, and angiogenesis of CRC [119]. In contrast, an antiangiogenic role has been demonstrated for circular FNDC3B, which has been found to be involved in microvesicles secreted by CRC cells and suppresses angiogenesis by directly regulating miR-937-5p to induce the expression level of the tumour-suppressor TIMP3, which inhibits the angiogenesis of CRC [120]. Other anti-angiogenic exosomal miRNAs have been described, such as miR-126, miR-125a-3p and miR-125a-5p, which target different regulators in CRC [121]. Both pro- and antiangiogenic exosomes provide predictive biomarkers for antiangiogenic treatment.

Recently, a murine model of colorectal cancer was used to explore whether TEX-miR-34a can serve as a favourable therapeutic option in CRC combinational therapies, one function of which is inhibiting angiogenesis by targeting VEGF [122]. The results of this study were promising and suggested that exosomes can be used as a treatment for cancer.

### Hepatic cancer

Exosomes participate in many mechanisms that promote hepatic tumour angiogenesis. Existing literature has indicated that the lncRNA H19, released via exosomes from CD90 + liver cancer cells, increases the expression of VEGF and the production of VEGF-R1, hence enhancing angiogenesis [123]. A large body of evidence has documented that exosomal microRNA-32-5p increases angiogenesis by activating the PTEN/PI3K/Akt pathway [124]. Moreover, miR-210 transferred by exosomes enhanced angiogenesis by directly repressing the expression of SNAD4 and STAT6 [125]. A series of experiments have been performed to show that miR-155 mediates angiogenic activity via exosomes under hypoxia and that it may be associated with proangiogenic factors [126]. Hepatocellular carcinoma (HCC) cell-derived exosomal miRNA-21 directly targeted PTEN (gene of phosphate and tension homology deleted on chromosome ten), leading to the activation of PDK1/AKT signalling in Hepatic stellate cells (HSCs), which are then transformed into CAFs [127]. Activated CAFs further promote cancer angiogenesis by secreting angiogenic cytokines, including VEGF, MMP2, MMP9, bFGF and TGF-β [128]. Furthmore, miR-21 can also shape a vascular microenvironment for HCC via the STAT3/VEGF signalling pathway, and miR-221 can activate the SAND/NF-κB pathway to upregulate the expression of CXCL16, which is an angiogenic factor [129]. Lysyl oxidase-like 1–4 (LOXL1–4), which was secreted by HCC-derived exosomes in a paracrine mechanism, was also reported to promote angiogenesis [130]. Exosomal circRNA-100,338 was found to be upregulated and could increase angiogenesis of HCC cells [131]. Moreover, circRNA-100,338 might decrease the expression of VE-cadherin and ZO-1 in HUEVCs to promote vascular endothelial cell permeability [131]. A pro‑angiogenic role has also been demonstrated for angiopoietin-2 (ANGPT2), which has been found to be contained in exosomes secreted by HCC cells and activates the Tie2-independent, AKT/eNOS and AKT/β-catenin pathways. [132] Research has shown that HCC cell-derived exosomes carrying LINC00161 activate the ROCK2 signalling pathway by inhibiting miR-590-3p and strengthen the tube-forming ability of HUVECs [133]. Hiroshi Yukawa et al. verified that HepG2-exosomes activated lumen formation by HUVECs [134]. Exosomes contain both upregulated and downregulated miRNAs, but their detailed influence is unknown [134]. Shihua Wang et al. found that HCC cell HepG2-derived exosomes could activate various kinases, such as AKT, STAT5α, GSK3 alpha/beta, p38 alpha, and ERK1/2, as well as the NF-κB signalling pathway in adipocytes to promote tube formation [135]. Recent experimental evidence has highlighted the role of exosomal microRNA-378b in HCC. HepG2 cell-derived exosomal miR-378b enhanced HCC cell angiogenesis by increasing MMP9, FGF2 and VEGFA expression, which may be linked with TGFBR3 [136]. Exosomal miR-1290 enhanced tube formation by directly targeting SMEK1 to alleviate the suppression of VEGFR2 phosphorylation [137]. In addition, HCC-cell-derived exosomes can be used as carriers to deliver CircCMTM3 to HUVECs. CircCMTM3 regulates SOX9 expression in HUVECs by sponging miR‐3619‐5p, which promotes angiogenesis and HCC cell tumorigenesis [138]. Moreover, exosome-delivered small nucleolar RNA host gene 16 (SNHG16) regulates GALNT1 expression by sponging miR-4500 via the PI3K/Akt/mTOR pathway to activate the formation of new HCC blood vessels, thus promoting the progression of HCC [139]. Exosomal RAB13, a potential regulator of HCC metastasis, was also associated with VEGF levels, microvessel density, and tube formation by vascular endothelial cells in both in vitro and in vivo models, suggesting that it promotes angiogenesis [140]. Moreover, HCC cell-derived exosomes containing C-Type Lectin Domain Family 3 Member B (CLEC3B) were able to inhibit the angiogenic ability of HMVECs via the repression of VEGF by activating AMPK signalling [141]. By targeting the transcription factor ERG (erythroblast transformation‑specific (ETS)‑related gene), exosomal miR-200b-3p plays a negative role in angiogenesis [142]. Similarly, exosomal miR-3682-3p targeted angiopoietin-1 (ANGPT1) via RAS-MEK1/2-ERK1/2 signalling, and attenuated angiogenesis [143]. All of these findings may indicate potential therapeutic targets for antiangiogenic therapy.

### Pancreatic cancer

Several studies have reported that exosomes released from pancreatic cancer (PC) cells contribute to enhancing cancer angiogenesis. Emanuela Pessolano et al. found that exosome-related annexin A1 (ANXA1) could induce angiogenesis in PC [144], but the mechanism remains unknown. PC cell-derived exosomal miR-27a, along with regulated B‐cell translocation gene 2 (BTG2), enhanced tumour angiogenesis [145]. LncRNA SNHG11 (SNHG11) has also been found to be highly expressed in the serum of PC patients, and is carried by exosomes. Mechanistically, SNHG11 acts as a ceRNA for miR‐324‐3p, therefore upregulating VEGFA, which has previously been implicated in tumour angiogenesis [146]. Environmental factors, such as hypoxia, play a prominent role in tumour angiogenesis [147]. The lncRNA UCA1, acting as a sponge of miR-96-5p, alleviated the repressive effects of miR-96-5p on the expression of its target gene AMOTL2 to enhance angiogenesis [148]. MiR-30b-5p was significantly enriched in hypoxic pancreatic ductal adenocarcinoma (PDAC) cell-derived exosomes, which could be transferred to HUVECs, resulting in the upregulation of tube formation and endothelial cell migration via downregulation of the gap junction protein GJA1 [149]. Emerging studies have proposed that hypoxia-induced miR-210 facilitates tumour angiogenesis and cellular permeability in PDAC, negatively regulating EFNA3 expression and participating in the PI3K/AKT/VEGFA or Wnt/β-catenin/RHOA pathways [150]. In contrast, miR-29b-containing exosomes in PC cells reduced angiogenesis migration and tube formation of HUVECs by targeting ROBO1 and SRGAP2 [151].

In addition to tumour cells, tumour-associated macrophages (TAMs) have recently been reported to enhance the angiogenic ability of endothelial cells. For example, Yin et al. experimented with TAM-derived exosomes, which are highly enriched in miRNA-501-3p, showing that incubating human microvascular endothelial cells with these exosomes promotes angiogenesis [152]. This effect may be related to the upregulation of VEGFA, VEGFR2, ANG2, and PLGF at the protein level and the enhancement of the angiogenic ability of human microvascular endothelial cells in vitro [152]. TAMs also overexpress miRNAs 155-5p and 221-5p [153]. These miRNAs can be secreted by exosomes and then delivered to endothelial cells to enhance tube formation ability, which is mediated by downregulation of the transcription factor E2F2 [153].

**Fig. 2:**
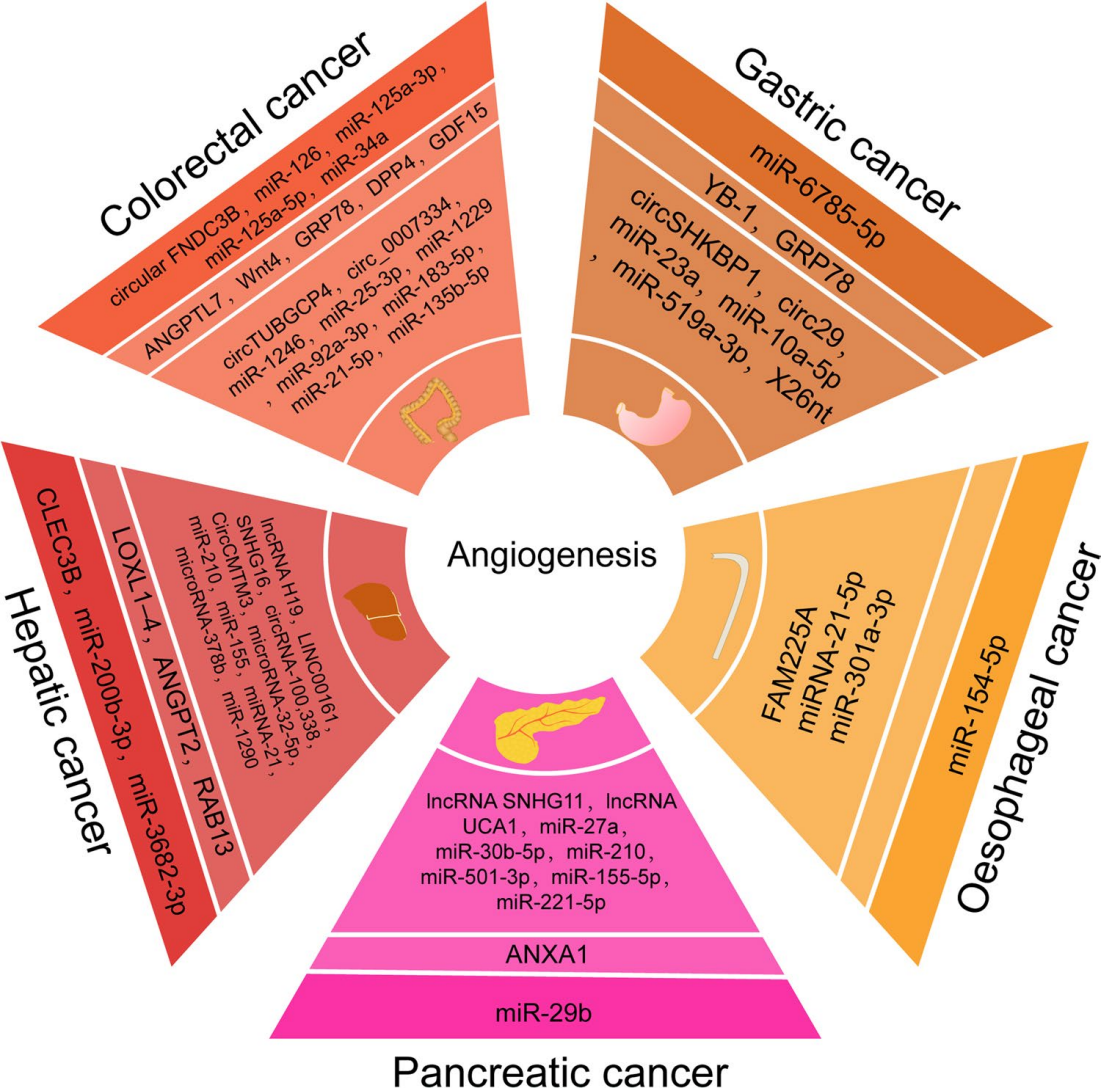
Exosomes in angiogenesis of digestive system tumours. The two light colored circles are factors of proangiogenesis. The first inner circle is protein; the outer circle is RNA. The factors in outermost dark circle are antiangiogenesis

Although many exosomes have been identified, as described above (Fig. 2), many are yet to be discovered and may have therapeutic potential.

### Anti-angiogenic therapy

As mentioned above, angiogenesis is one of the most important processes for tumour development. Therefore, antiangiogenic therapy (AAT) has attracted attention, and many drugs are being studied [154–156]. Four main strategies are considered to develop antiangiogenic agents: inhibiting endogenous factors promoting angiogenesis, identifying and applying natural angiogenesis inhibitors, inhibiting the molecules that promote the invasion of the surrounding tissue through tumour blood vessels, and incapacitating actively proliferating endothelial cells [53]. Bevacizumab, ramucirumab and aflibercept, which are antiangiogenic agents, are indicated for most colorectal cancer patients [157]. Clinical trials have certified that sorafenib and lenvatinib are multikinase inhibitors, regorafenib and cabozantinib are tyrosine kinase inhibitors, and ramucirumab is a monoclonal antibody, and all of them target the factors of the VEGF axis to block tube formation in hepatocellular cancer [158]. Several preclinical and clinical studies have demonstrated the efficacy of anti-VEGF-A therapies in pancreatic cancer, and some agents have emerged, such as bevacizumab and sunitinib [159]. Any drug has side effects [160] and prolonged treatment will ultimately lead to resistance. The main mechanisms of antiangiogenic drug-related resistance involve revascularization, tumour vasculature protection, accentuated invasiveness of tumour cells, and increased metastasis through different modes of vascularization [161]. Therefore, novel antiangiogenic strategies, the combination of antiangiogenesis agents and chemotherapy or immunotherapy, have been developed [160].

In recent years, nanotechnology-based theranostic strategies have gained great interest for their unique properties or delivery of antiangiogenic agents to tumour sites by active (ligand-mediated) or passive (enhanced EPR effect of antiangiogenic agents to the tumour site) targeting [162]. Although many nanoparticles employed for the transmission of antiangiogenic agents have been studied, such as cerium oxide, gold, silver, copper, silicate, carbon, and peptide-conjugated nanoparticles, only 0.7% of the administered nanoparticles are considered to reach the targeted site [163]. Although nanoparticles have shown great potential in biology and medicine for their wide range of applications, their potential toxicity, non-specific uptake and clearance, as well as the conditions during their production, currently limit their use in these fields. Many experiments in animal models and clinical studies are ongoing to prove that the nanomedicine approach is feasible as a new antiangiogenic treatment [164].

### Exosomes in anti-angiogenic therapy

With the advancement of 3D culture models, it is now possible to artificially manipulate the cell architecture by introducing various components, including collagen, fibronectin, and cytokines [165]. These alterations have the potential to induce variations in the content and cargo of exosomes [166, 167]. Consequently, the manipulation of the cellular environment using 3D culture models presents novel prospects for comprehending and harnessing exosomes as biomarkers in the realms of diagnosis and treatment [168]. Exosomes carry unique substances in distinct cancers and their levels reflect the clinical staging and prognosis to some degree [169–172]. At the same time, the transfection or knockdown of exosomes can have opposing effects on cancer cells [173]. For example, exosome-encapsulated hepatocyte growth factor (HGF) siRNA could exert inhibitory effects on the proliferation and migration of vascular cells via the repression of HGF and VEGF [174]. A recent experiment with liver cancer HepaRG cells treated with camel milk exosomes resulted in a significant downregulation of VEGF [175], and another antiangiogenic method, apatinib monotherapy, showed a reduction in exosome secretion in colorectal cancer cells [176]. Thus, exosomes have enormous therapeutic potential.

With in-depth research, exosomes have been confirmed to affect antiangiogenic drug-related resistance. Ying Gao et al. used both in vivo and in vitro assays to conclude that exosomal miR-494-3p derived from Golgi phosphoprotein 3 (GOLPH3)-overexpressing HCC cells promoted the angiogenesis ability of HUVECs and induced sorafenib resistance in HCC cells, which has important potential clinical value in improving therapeutic efficiency in HCC patients [177].

In addition to being targets, exosomes can be carriers. Compared with synthetic nanoparticles, exosomes are specific, safe, of cell-origin, and natural carriers that have a long half-life and nonimmunogenic properties for drug delivery systems [178–180]. Specifically, exosomes have a lipid bilayer and a hydrophilic core; thus, they can transmit both lipid- and water-soluble drugs [181]. Exosome-based nanocarriers can be designed in direct or indirect engineering processes [182]. Notably, some researchers have attempted to engineer exosomes in the antiangiogenic treatment of ovarian cancer, alone or combined with apatinib [183]. In a similar case, some studies have shown that anticancer drug DOX bound to exosomes plays roles in lung cancer or colorectal cancer cells, and studies have shown that exosomes can affect the central nervous system (CNS) as carriers of proteins and RNAs [184].

## Conclusion and future perspectives

Malignant tumours of the digestive system occur in daily life. Angiogenesis is an important process in tumour development that is controlled by the balance of pro- and anti-angiogenic factors. Exosomes have a marked effect. To date, a multitude of exosomes carrying mRNA/lncRNA/protein have been discovered. Correspondingly, directly targeting them by enhancing their secretion or decreasing their expression is an anti-angiogenic therapeutic strategy. Alternatively, exosomes can be used as delivery vehicles for drugs that can promote or inhibit angiogenesis. Simultaneously, exosome functions have recently attracted researchers’ attention, especially regarding angiogenesis. However, the mechanism by which exosomes selectively package their cargo remains unclear. This limitation has led to the exploration of improving treatments, such as chemotherapy, immunotherapy, or combination therapy [185]. Advancements in technology will advance the use of exosomes in the digestive system.

However, the clinical application of exosomes remains subject to limitations. The methods used to isolate, purify, and identify exosomes in the laboratory cannot be used in clinical research because of inconvenience. Firstly, the extraction process of exosomes is relatively complicated, requiring multiple methods such as ultracentrifugation, precipitation, ultrafiltration, and so on. The operation process is also quite cumbersome. Secondly, the number of exosomes extracted is usually small, and we need large volumes of body fluids to obtain sufficient exosomes to meet clinical requirements. Additionally, the identification of exosomes is also relatively complex, necessitating methods such as transmission electron microscopy, particle size analysis, protein markers, etc., to ensure their purity and biological activity [186]. Therefore, improving the efficiency of exosome isolation methods and developing strategies to obtain a larger yield of exosomes from limited fluid sources is necessary to facilitate their clinical translation. As a result, the application of exosomes in the diagnosis and therapy of cancer remains difficult. Exosomes will be widely used shortly.

## Data Availability

Data sharing is not applicable to this article as no datasets were generated or analysed during the current study.

## Abbreviations

AAT: Antiangiogenic therapy
ADO: Adenosine
ANGPT1: Angiopoietin-1
ANGPT2: Angiopoietin-2
ANGPTL7: Angiopoietin-like 7
ANXA1: Annexin A1
ARF6: ADP ribosylation factor 6
BTG2: B‐cell translocation gene 2
CA125: Carbohydrate antigen 125
CA19-9: Carbohydrate antigen 19–9
CAF: Cancer-associated fibroblast
CEA: Carcinoembryonic antigen
CLDN11: Claudin-11
CLEC3B: C-Type Lectin Domain Family 3 Member B
CNS: Central nervous system
CRC: Colorectal cancer
DACT2: Disheveled binding antagonist of beta-catenin 2
Dkk-3: Dickkopf-3
DPP4: Dipeptidyl peptidase IV
EC: Endothelial cell
ECM: Extracellular matrix
ERG: Erythroblast transformation specific (ETS) related gene
ESCC: Oesophageal squamous cell carcinoma
ESCRT: Endosomal sorting complex needed for transport
ESE: Early-sorting endosome
FGF: Fibroblast growth factor
FOXM1D: Forkhead Box M1 D
Foxo: Forkhead Box O
GC: Gastric cancer
GDF15: Growth/differentiation factor 15
GOLPH3: Golgi phosphoprotein 3
GRP78: Glucose-regulated protein 78
HCC: Hepatocellular carcinoma
HGF: Hepatocyte growth factor
HK2: Hexokinase 2
HSCs: Hepatic stellate cells
HUVECs: Human umbilical vein endothelial cells
IA: Intussusceptive angiogenesis
ILVs: Intraluminal vesicle
IRE1: Inositol- requiring enzyme 1 alpha
KIF14: Kinesin family member 14
KLF12: Krüppel-like factor 12
KLF2: Krüppel-like factor 2
KLF4: Krüppel-like factor 4
KRIT1: Krev interaction trapped protein 1
LncRNAs: Long noncoding RNAs
LOXL1–4: Lysyl oxidase-like 1–4
LSE: Late-sorting endosome
MLL3: Histone-lysine N-methyltransferase-3
MMP: Matrix metalloproteinase
MVBs: Multivesicular bodies
NSF: N-ethyl- maleimide-sensitive factor
PAR1: Protease-activated receptor-1
PABPC1: Poly (A) binding protein cytoplasmic 1
PC: Pancreatic cancer
PDAC: Pancreatic ductal adenocarcinoma
PDGF: Platelet-derived growth factor
PKM2: Pyruvate kinase M2
PLD2: Phospholipase D2
PLGF: Placental growth factor
PML: Promyelocytic leukaemia
POSTN: Periostin
PTEN: Phosphatase and tensin homologue
SA: Sprouting angiogenesis
sEVs: Small extracellular vesicles
SNARE: Soluble N-ethyl- maleimide-sensitive factor-attachment protein receptor complex
SNHG16: Small nucleolar RNA host gene 16
SYT7: Synaptotagmin 7
TAMs: Tumour-associated macrophages
TEX: Tumour-derived exosomes
TME: Tumour microenvironment
TSP-1: Thrombospondin-1
TXNIP: Thioredoxin-interacting protein
VC: Vessel co-option
VEGF-A: Vascular endothelial growth factor A
VM: Vascular mimicry
ZMYND11: Zinc finger MYND-type containing 11
